# Response of Cytoprotective and Detoxifying Proteins to Vanadate and/or Magnesium in the Rat Liver: The Nrf2-Keap1 System

**DOI:** 10.1155/2021/8447456

**Published:** 2021-12-13

**Authors:** Agnieszka Ścibior, Iwona Wojda, Ewa Wnuk, Łukasz Pietrzyk, Zbigniew Plewa

**Affiliations:** ^1^Laboratory of Oxidative Stress, Centre for Interdisciplinary Research, The John Paul II Catholic University of Lublin, Poland; ^2^Department of Immunobiology, Institute of Biological Sciences, Maria Curie-Sklodowska University (UMCS), Lublin, Poland; ^3^Chair of Anatomy, Department of Didactics and Medical Simulation, Medical University of Lublin, Poland; ^4^Department of General, Oncological, and Minimally Invasive Surgery, 1 Military Clinical Hospital with The Outpatient Clinic in Lublin, Poland

## Abstract

Oxidative stress (OS) is a mechanism underlying metal-induced toxicity. As a redox-active element, vanadium (V) can act as a strong prooxidant and generate OS at certain levels. It can also attenuate the antioxidant barrier and intensify lipid peroxidation (LPO). The prooxidant potential of V reflected in enhanced LPO, demonstrated by us previously in the rat liver, prompted us to analyze the response of the nuclear factor erythroid-derived 2-related factor 2/Kelch-like ECH-associated protein 1 (Nrf2-Keap1) system involved in cellular regulation of OS to administration of sodium metavanadate (SMV, 0.125 mg V/mL) and/or magnesium sulfate (MS, 0.06 mg Mg/mL). The levels of some Nrf2-dependent cytoprotective and detoxifying proteins, i.e., glutathione peroxidase (GPx), glutathione reductase (GR), glutathione S-transferase (GST), glutamate cysteine ligase catalytic subunit (GCLC), glutathione synthetase (GSS), NAD(P) H dehydrogenase quinone 1 (NQO1), UDP-glucumno-syltransferase 1 (UGT1), and heme oxygenase 1 (HO-1); glutathione (GSH); metallothionein (MT1); and glutamate-cysteine ligase (GCL) mRNA were measured. We also focused on the V-Mg interactive effects and trends toward interactive action as well as relationships between the examined indices. The elevated levels of Nrf2, GCL mRNA, and GCL catalytic subunit (GCLC) confirm OS in response to SMV and point to the capacity to synthesize GSH. The results also suggest a limitation of the second step in GSH synthesis reflected by the unchanged glutathione synthetase (GSS) and GSH levels. The positive correlations between certain cytoprotective/detoxifying proteins (which showed increasing trends during the SMV and/or MS administration, compared to the control) and between them and malondialdehyde (MDA), the hepatic V concentration/total content, and/or V dose (discussed by us previously) point to cooperation between the components of antioxidant defense in the conditions of the hepatic V accumulation and SMV-induced LPO intensification. The V-Mg interactive effect and trend are involved in changes in Nrf2 and UGT1, respectively. The p62 protein has to be determined in the context of potential inhibition of degradation of Keap1, which showed a visible upward trend, in comparison with the control. The impact of Mg on MT1 deserves further exploration.

## 1. Introduction

Vanadium (V) is a transition element released into the environment from both natural sources and anthropogenic activity [1]. It may accumulate in the environmental media [2, 3] and in tissues and organs of living organisms [1]. At certain levels, V is toxic and can lead to serious health problems, summarized in our previous reports [1, 4]. Bearing these facts in mind, there is a need for detailed and systemic investigations to evaluate the toxicity of V and possible toxic effects caused by its chronic treatment and to recognize the consequences of possible interactions of V with elements having antioxidant properties, including magnesium (Mg), which is important in view of the strong prooxidant potential of V.

Several *in vitro* and *in vivo* studies have shown that, in some conditions, V can act as a strong prooxidant and generate oxidative stress (OS) [5, 6], which is well-known to lead to permanent cell and tissue damage and, consequently, to initiation of the disease process [7]. Vanadium may also disrupt the antioxidant barrier and intensify lipid peroxidation (LPO) [5].

Our previous studies conducted on a rodent model demonstrated that V administered as sodium metavanadate (SMV) led to an increase in the level of malondialdehyde (MDA) in rat erythrocytes (RBC) [8], kidney [9], and liver [10, 11]. It also attenuated antioxidant defense mechanisms in the rat RBC [12] and bone [13]. In addition, our studies showed that OS is involved in the mechanism underlying the development of SMV-induced functional renal disorders [14].

The results of our *in vivo* studies, which revealed the prooxidant potential of V reflected in the enhanced LPO in the rat hepatic tissue after 12-week administration of SMV alone and in combination with magnesium sulfate (MS) [10], prompted us to explore mechanisms associated with OS. We intended to recognize the role of the nuclear factor erythroid-derived 2-related factor 2 (Nrf2), which directly affects the reactive oxygen species (ROS) homeostasis and induces the expression of defensive and detoxifying genes in response to OS [15–18]. Besides Nrf2 and its negative regulator, i.e., Kelch-like ECH-associated protein 1 (Keap1), which tightly controls the Nrf2 cellular function [19, 20] and acts as a sensor of disturbances in cellular homeostasis [15, 16], we determined certain nonenzymatic markers of OS and some Nrf2-related cytoprotective and detoxifying enzymes in the rat liver, i.e., (a) the glutamate cysteine ligase catalytic subunit (GCLC) and glutathione synthetase (GSS), the induction of which is a key step in the defense mechanism, as they are involved in the synthesis of glutathione (GSH) that plays a pivotal role in reducing oxidative damage [15]; (b) glutathione peroxidase (GPx) and glutathione reductase (GR) responsible for the GSH redox cycle [21, 22]; (c) NAD(P)H dehydrogenase quinone 1 (NQO1), glutathione S-transferase (GST), UDP-glucumno-syltransferase 1 (UGT1), and heme oxygenase-1 (HO-1), which play a prominent role in cellular adaptation to many stress conditions, including OS [20, 21, 23–28]; and (d) metallothionein-1 (MT1), as known metallothioneins (MTs) are capable of detoxifying excessive amounts of transition elements, thus protecting against their toxicity and oxidative injury [29, 30]. The hepatic glutamate cysteine ligase (GCL) expression in response to SMV and MS was investigated as well. In addition, we focused on the vanadium-magnesium (VxMg) interactive effects and trends toward interactive action and their potential impact on the parameters explored.

To the best of our knowledge, no rodent-model studies have been conducted before with respect to the assessment of the Nrf2-mediated defense mechanisms against OS in the liver of rats receiving SMV (0.125 mg V/mL) separately and simultaneously with MS (0.06 mg Mg/mL). No *in vivo* individual study on the consequence of possible VxMg interactive effects on the key player in antioxidant defense and its repressor and on the hepatic glutamate-cysteine ligase (GCL) mRNA, Nrf2-dependent cytoprotective/detoxifying proteins, and MT1 has been carried out either. The present report is also the first to reveal many relationships between the above-mentioned indices examined in the rat hepatic tissue under the influence of SMV and/or MS.

## 2. Material and Methods

### 2.1. Reagents

Sodium metavanadate (NaVO_3_), magnesium sulfate (MgSO_4_), a cleaning agent for removing RNase (RNaseZAP), and a kit for RNA extraction providing a simple and convenient way to isolate total RNA from mammalian cells and tissues (RTN70-1KT, GenElute Mammalian Total RNA Miniprep Kit) were obtained from Sigma Chemical (St. Louis, USA). The enzyme-linked immunosorbent assay (ELISA) kits for rat nuclear factor erythroid-derived 2-related factor 2 (Nrf2, ELISA kit No. QY-E11823), rat Kelch-like ECH-associated protein 1 (Keap1, ELISA kit No. QY-E11822), rat glutathione (GSH, ELISA kit No. QY-E11731), rat metallothionein-1 (MT1, ELISA kit No. QY-E10241), rat glutamate cysteine ligase catalytic subunit (GCLC, ELISA kit No. QY-E11835), rat glutathione synthetase (GSS, ELISA kit No. QY-E11907), rat glutathione reductase (GR, ELISA kit No. QY-E11828), rat glutathione peroxidase (GPx, ELISA kit No. QY-E11657), rat glutathione S-transferase (GST, ELISA kit No. QY-E11772), rat UDP-glucumno-syltransferase 1 (UGT1, ELISA kit No. QY-E11832), rat NAD(P)H dehydrogenase quinone 1 (NQO1, ELISA kit No. QY-E11831), and rat heme oxygenase 1 (HO-1, ELISA kit No. QY-E) were acquired from Qayee Bio-Technology (Shanghai, China). In turn, a kit for the removal of possible DNA contamination from extracted RNA (Turbo DNA-free kit, Cat. No. AM1907), a kit for the reverse transcription reaction (RT) (High Capacity cDNA Reverse Transcription kit with RNase inhibitor, Cat. No. 4374966, Applied Biosystems), the Power SYBR Green polymerase chain reaction (PCR) Master Mix (Cat. No. 4368577, Applied Biosystems), the nuclease-free water not diethylpyrocarbonate- (DEPC-) treated (AM9939), and Dulbecco's Phosphate-Buffered Saline (DPBS, no Ca/no Mg, pH = 7 − 7.3) were purchased from Thermo Fisher Scientific. Freeze-drying oligonucleotides (GCL and Sdha) were obtained from GenoMed (Poland). All the chemicals were of the highest quality available.

### 2.2. Instrumentation

A deep-freezer HFU 486 Basic^1^ (Thermo Fisher Scientific, Germany); an XA 100 3Y.A^1^ analytical balance (Radawag, Poland); and a BioGen PRO200 homogenizer^1^ (ProScientific, USA) were used to prepare the hepatic samples for determination of the selected biochemical parameters and for RNA isolation procedure, whereas a Polwater DL-100 V717^1^ (Labopol, Poland) was used to prepare some reagents for the ELISA Assay. A MiniSpin Plus microcentrifuge^1^ (Eppendorf, Germany), a thermomixer 5355-comfort^1^ (Eppendorf, Germany), and a C 1000 thermal cycler^1^ with a 48/48 dual fast reaction module-gradient (Bio Rad, USA) were used in the reverse transcription reaction. A double beam spectrophotometer U-2900^1^ (Hitachi, Japan) was used to measure the concentration and purity of the isolated material (RNA), and StepOnePlus Engine (Applied Biosystems) was used for real-time PCR. In turn, a Synergy 2 multimode microplate reader (BioTek Instruments Inc., Winooski, VT, USA) with an ELMI DTS-4 digital thermostatic microplate shaker^1^ (ELMI SIA, Riga, Latvia) and an ELx50 microplate strip washer^1^ (BioTek Instruments Inc., Winooski, VT, USA) were used to determine Nrf2, Keap1, GSH, MT1, GCLC, GSS, GR, GPx, GST, HO-1, UGT1, and NQO1 in the rat hepatic supernatants with the ELISA technique.

### 2.3. Animals and Experimental Protocol

Livers were obtained from some outbred albino male Wistar rats used in our previous experiments, in which all the animals were divided into 4 groups and maintained individually in stainless steel cages in a room with controlled conditions. During the whole experiment, all rats received (*ad libitum*) properly balanced standard granulated laboratory rodent chow (Labofeed B, Fodder and Concentrate Factory, Kcynia, Poland) and appropriate fluids to drink every day over a 12-week period in special bottles with a scale [31]: control—deionized water (12-15 rats/per group), SMV—sodium metavanadate (12-15 rats/per group) at a concentration of 0.125 mg V/mL (pH = 7.2), MS—magnesium sulfate (10-12 rats/per group) at a concentration of 0.06 mg Mg/mL (pH = 5.7), and SMV+MS—14-15 rats/per group at a concentration of 0.125 mg V + 0.06 mg Mg/mL (pH = 7.1). As described previously [32], the intake of food, deionized water, and the SMV, MS, and SMV+MS water solutions was monitored daily throughout the experimental period, and body weight was checked weekly. After 12 weeks, the livers were harvested immediately after sectioning and directly rinsed in ice-cold physiological saline solution (0.9% NaCl). Then, the wet weights were recorded, and the samples were stored at -80°C until the planned analyses. The experiment was conducted in accordance with the experimental protocol approved by the 1^st^ Local Ethical Committee for Animal Studies in Lublin [31].

The concentrations of SMV and MS were selected based on previous studies in a rodent model [33–39]. In addition, we took into consideration the report of the Dietary Reference Intakes [40], in which diarrhea was mentioned as the most sensitive toxic manifestation of excess Mg intake. Therefore, the amount of MS in drinking water was chosen by us to be not too high. In our experimental conditions, a 10 mg higher Mg concentration (60 mg Mg/L) was used from the Maximal Admissible Concentration of this mineral for potable water in Poland [41]. Moreover, we took into consideration the reports by Kučera et al. [42] and Lees [43] in which the concentrations of V in the blood and urine of occupationally exposed people are comparable with those observed in our experimental conditions in the SMV-exposed rats [14, 32]. The reasons for which V and Mg were selected for testing in *in vivo* conditions (in a rodent model) during separate and combined administration were provided previously [44]. As highlighted, the potential protective influence of Mg on limiting of the toxic V action and the possible interactions of V (as a prooxidant) with Mg (as an antioxidant) were the subject of our special interest in research on these elements in an *in vivo* model.

As already presented [32], the fluid and food intake as well as body weight significantly decreased in the rats receiving SMV separately and in combination with MS, compared to the control and MS-supplemented animals; the two-way ANOVA analysis suggested that all these changes in the rats exposed to SMV during the MS supplementation were only influenced by the independent V action. Daily V and Mg doses consumed by the SMV-, MS-, and SMV+MS-treated rats, estimated on the basis of the 24-consumption of the SMV, MS, and SMV+MS solutions administered to rats in drinking water, were as follows: ~13 mg V/kg b.wt./24 h, 7.5 mg Mg/kg b.wt./24 h, and ~13 mg V/kg b.wt./24 h + 6.3 mg Mg/kg b.wt./24 h, respectively.

### 2.4. Preparation of Hepatic Supernatants for Analyses with the ELISA Technique

First, livers frozen at -80°C were thawed at room temperature. Then, appropriate portions of the organ were cut and thoroughly washed in DPBS. After draining with tissue paper, the hepatic samples were inserted into Eppendorf tubes, homogenized in DPBS, and centrifuged with cooling (3000 rpm, 15 min, 4°C). The liver supernatants were used to measure the selected parameters with commercial rat-specific ELISA kits.

#### 2.4.1. ELISA Assay

Quantitative sandwich ELISA kits were used to determine the hepatic levels of Nrf2, Keap1, GSH, MT1, GCLC, GSS, GR, GPx, GST, HO-1, UGT1, and NQO1. All analyses were carried out in strict accordance with the manufacturer's recommendations. The standard curves were created by plotting absorbance vs. concentration. Data were interpolated from the standard curves to calculate the amount of Nrf2, Keap1, GSH, MT1, GCLC, GSS, GR, GPx, GST, HO-1, UGT1, and NQO1. The results were expressed per g of the fresh liver weight.

### 2.5. RNA Extraction and RT

For RNA extraction, 40 mg of frozen rat liver was used. The weighed tissue was placed in 500 *μ*L of lysis solution, and then, the extraction was performed according to the manufacturer's protocol. The next step was the removal of eventual DNA contamination from extracted RNA with the use of DNase. After the isolation, the concentration and purity of RNA were measured using absorption coefficients A_260_/A_280_. RNA was stored at -20°C.

The RT reaction was performed according to the manufacturer's instruction on 20 *μ*L of sample. One microgram of total RNA was used as a template, and random hexamer primers were employed. The samples were placed into the thermocycler and subjected to the reaction in conditions proposed by the manufacturer: step 1: 25°C, 10 min; step 2: 37°C, 120 min; step 3: 85°C, 5 min; step 4: 4°C, ∞. The cDNA obtained was stored at -20°C.

### 2.6. Real-Time PCR

For determination of the relative expression level of GCL, quantitative real-time PCR was performed with the use of a 100 times diluted mixture after reverse transcription. The reaction conditions with the use of Power SYBR Green PCR Master Mix were as follows: 95°C, 10 min, 40x (95°C 15 sec, 60°C 1 min—annealing and extension). The primers for the Sdh housekeeping gene (encoding the succinate dehydrogenase complex) were as follows: forward 5′-TGGTCACTCGGGCTGGTT-3′; reverse: 5′-CGGCACCCTTCTGTGATGA-3′. The number of transcripts for this gene did not change in the experimental conditions.

The primers for GCL were as follows: forward 5′-GGAGGAACGATGTCCGAGTTC-3′; reverse: 5′-TCGTGCAAAGAGCCTGATGT. The results were normalized to the housekeeping gene (*Sdh*), taking into consideration the efficiency of reaction with both sets of primers [45, 46].

### 2.7. Statistical Analysis

The statistical analysis was performed using the statistical software SPSS (IBM SPSS Statistics, Version 26) and Statistica (StatSoft, Version 12). Standardization of variables was performed prior to the analysis. The distribution patterns in the data and the homogeneity of variances were verified employing Shapiro-Wilk's normality test and Levene's test, respectively. Hartley's Fmax, Cochran's C, and Bartlett's tests were carried out when the hypothesis of equal variances was rejected by Levene's test. Normally distributed data were compared *via* post hoc *analysis* (Tukey's HSD or T3 Dunnett's tests) of the significant ANOVA results. The two-way analysis of variance (two-way ANOVA) with V and Mg factors and the *F* test were employed to indicate the main effects of V and Mg and the VxMg interactive effects on the analyzed parameters. The differences were considered significant at *P* lower than 0.05. Pearson's correlation analysis was performed to assess the relationships among measurable variables. The correlations were considered significant at *P* < 0.05. In addition, a simple linear regression analysis was used to determine the relationships between certain variables and the doses of vanadium and magnesium (predictors) consumed by the rats with drinking water during the whole experiment. *P* < 0.05 was considered statistically significant, and only significant effects were presented in the report. The results are expressed as the mean with the standard error of the mean (SEM) and standard deviation (SD).

## 3. Results

### 3.1. Hepatic Levels of Nrf2 and Keap1

**Figure 1:**
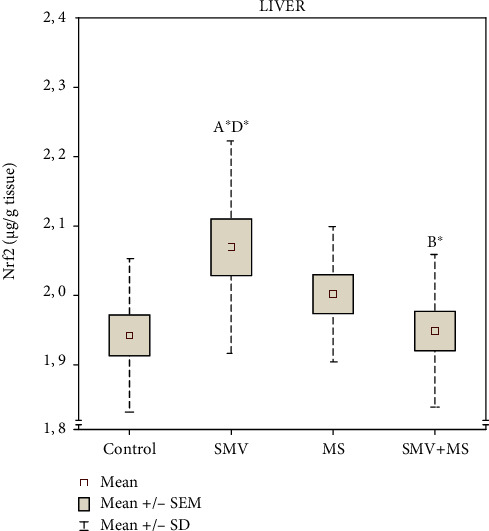
The concentration of nuclear factor erythroid-derived 2-related factor 2 (Nrf2) in the liver. Differences in the groups are indicated by the following: ^A^versus the control rats (Group I), ^B^versus the SMV-exposed rats (Group II), ^C^versus the MS-supplemented rats (Group III), and ^D^versus the SMV+MS cosupplied rats (Group IV) (the Tukey HSD test). ^∗^*P* < 0.05.

The amount of the Nrf2 protein in the liver of rats receiving SMV alone increased significantly, compared to the control and the SMV+MS cosupplied animals. In the MS-supplemented rats, no significant changes in the level of this protein were found. In turn, the hepatic amount of the Nrf2 protein in the rats exposed to SMV during the MS administration decreased markedly, compared to those receiving SMV alone, and this decrease was only influenced by the VxMg interaction, as suggested by the two-way ANOVA (Figure 1, Table 1).

**Figure 2:**
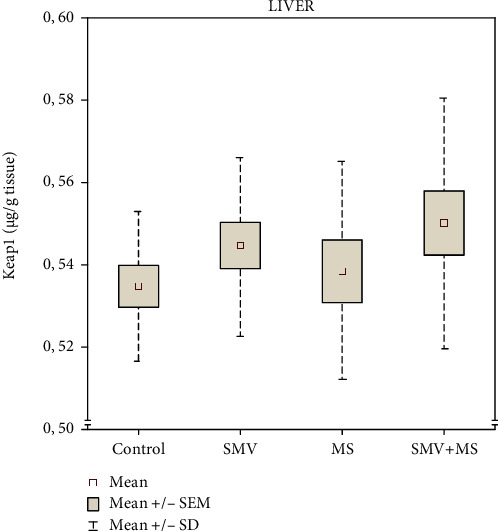
The concentration of Kelch-like ECH-associated protein 1 (Keap1) in the liver: control rats (Group I), SMV-exposed rats (Group II), MS-supplemented rats (Group III), and SMV+MS cosupplied rats (Group IV).

As far as Keap1 is concerned, its hepatic amount did not change significantly in the rats receiving SMV and MS separately as well as SMV and MS in combination; only an upward trend was observed, compared to the control (Figure 2). The two-way ANOVA revealed that the hepatic Keap1 level in rats exposed to SMV during the 12-week MS supplementation was not affected by either an independent action of V and Mg or by their mutual interaction (Table 1).

### 3.2. Hepatic GSH Level

**Figure 3:**
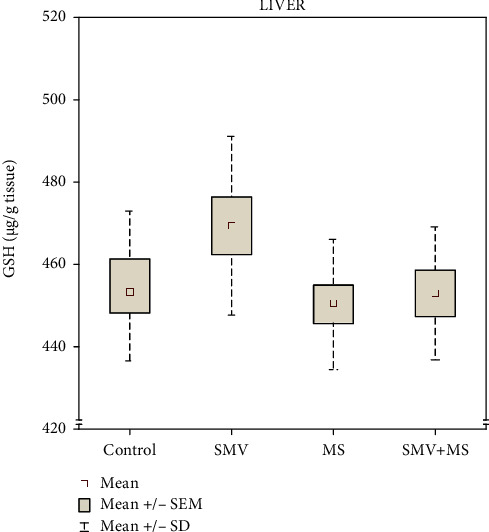
The concentration of reduced glutathione (GSH) in the liver: control rats (Group I), SMV-exposed rats (Group II), MS-supplemented rats (Group III), and SMV+MS cosupplied rats (Group IV).

The concentration of GSH in the liver of rats receiving SMV and MS separately and in combination did not change markedly, compared to the control (Figure 3), and no significant V, Mg, or VxMg effects were indicated by the two-way ANOVA with respect to the level of this compound in the liver of the SMV+MS cosupplied rats (Table 1).

### 3.3. Hepatic Enzymes Involved in GSH Biosynthesis and GCL mRNA

**Figure 4:**
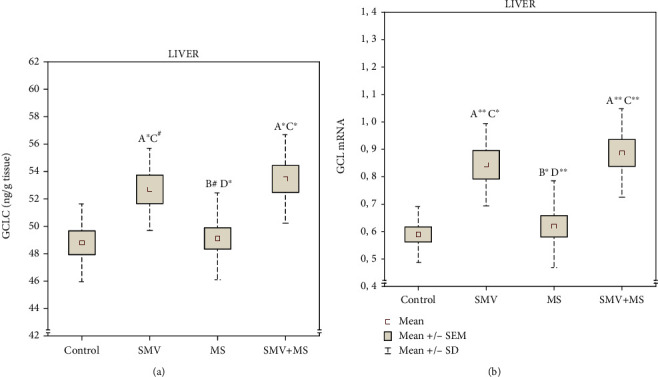
The level of (a) glutamate cysteine ligase catalytic subunit (GCLC)^1^ and (b) glutamate cysteine ligase (GCL) mRNA^2^ in the liver. Differences in the groups are indicated by the following: ^A^versus the control rats (Group I), ^B^versus the SMV-exposed rats (Group II), ^C^versus the MS-supplemented rats (Group III), and ^D^versus the SMV+MS cosupplied rats (Group IV) (the Tukey HSD test^1^ and the Dunnett T3 test^2^). ^∗^*P* < 0.05, ^∗∗^*P* < 0.01, and ^#^*P* = 0.065.

The level of GCLC in the liver of rats receiving SMV alone and together with MS significantly increased, compared to the control and MS-supplemented animals (Figure 4(a)). In the latter group, the hepatic GCLC level remained unchanged, compared to the control, but markedly decreased in comparison with the SMV-exposed and SMV+MS coadministered rats (Figure 4(a)). As suggested by the two-way ANOVA, the increase in the level of GCLC in the liver of the rats exposed to SMV during the MS supplementation was induced by the independent action of V only (Table 1).

As for the GCL expression, SMV alone and the SMV+MS combination significantly elevated the hepatic GCL mRNA level, compared to the control (by 43% and 51%, respectively) and the MS-supplemented animals (by 36% and 43%, respectively) (Figure 4(b)). In turn, MS alone did not significantly change the mRNA expression of GCL, compared to the control. In turn, GCL mRNA markedly decreased (by 26.5% and 30%, respectively) in comparison with the SMV-exposed rats and those receiving SMV together with MS (Figure 4(b)). The two-way ANOVA suggests that the changes in the hepatic GCL mRNA level in the SMV+MS coadministered rats might result from the independent action of V only (Table 1).

**Figure 5:**
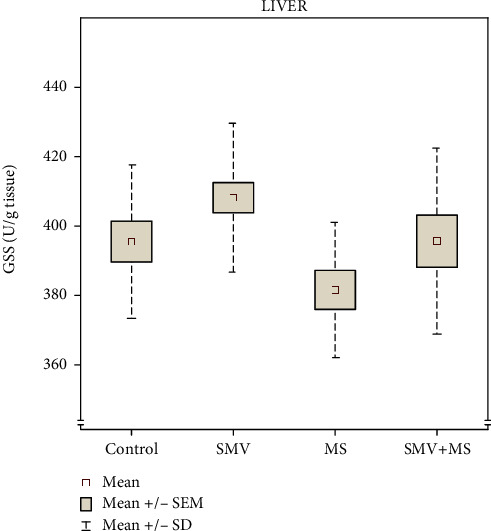
The level of glutathione synthetase (GSS) in the liver: control rats (Group I), SMV-exposed rats (Group II), MS-supplemented rats (Group III), and SMV+MS cosupplied rats (Group IV).

In the case of GSS, it should be highlighted that even though the post hoc comparisons did not show significant differences between the groups (Figure 5), the two-way ANOVA revealed trends toward an independent effect of V and Mg on the level of this enzyme in the liver of the SMV+MS cosupplied rats (Table 1). However, no interactive VxMg effect was indicated (Table 1).

### 3.4. Hepatic MT1 Level

**Figure 6:**
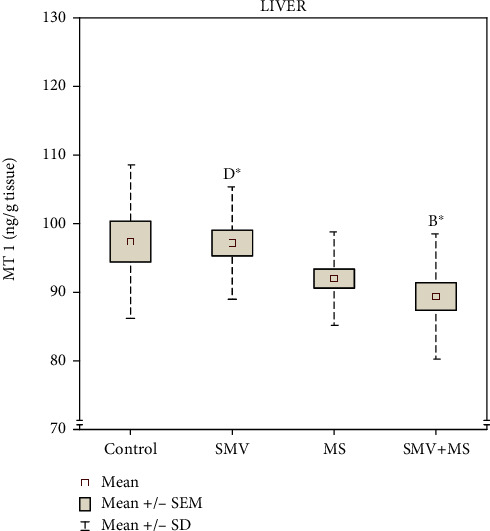
The concentration of metallothionein-1 (MT1) in the liver. Differences in the groups are indicated by the following: ^A^versus the control rats (Group I), ^B^versus the SMV-exposed rats (Group II), ^C^versus the MS-supplemented rats (Group III), and ^D^versus the SMV+MS cosupplied rats (Group IV) (the Dunnett T3 test). ^##^*P* = 0.058.

The concentration of MT1 in the liver of rats receiving SMV alone was at a similar level as in the control (Figure 6). The level of this protein in the liver of the MS-supplemented animals was also unaffected; however, it clearly tended to be lower, in comparison with the control. Similarly, in the SMV+MS coadministered rats, the hepatic MT1 concentration also tended to decrease, but these changes turned out to be statistically significant only in comparison with the SMV-exposed animals (Figure 6). The two-way ANOVA demonstrated that the decline in the hepatic MT1 level in the SMV+MS cosupplied rats was related to the independent action of Mg only (Table 1).

### 3.5. Hepatic Levels of GSH-Metabolizing Enzymes

The level of GR did not differ significantly between the four experimental groups. Only upward trends were observed, compared to the control (Figure S1). The highest GR level was found in the liver of the SMV+MS cosupplied rats (Figure S1), and as indicated by the two-way ANOVA, this increase was a consequence of the independent action of Mg only (Table S1). As for GPx, although the two-way ANOVA did not reveal any independent action of V, Mg, and/or the VxMg interaction (Table S1), there were visible upward trends in the level of GPx in the liver of the SMV and SMV+MS coadministered rats, compared to the control and the MS-supplemented animals (Figure S2). In the case of GST, a distinct upward trend in the level of this protein was observed in the SMV-exposed rats, but this difference did not turn out to be significant, compared to the control (Figure S3). Moreover, no significant differences were found in the hepatic GST level in the MS-treated rats and the SMV+MS cosupplied animals, in comparison with the control (Figure S3); no V, Mg, or VxMg effects were demonstrated by the two-way ANOVA (Table S1).

### 3.6. Hepatic Levels of Selected Cytoprotective/Detoxifying Enzymes

**Figure 7:**
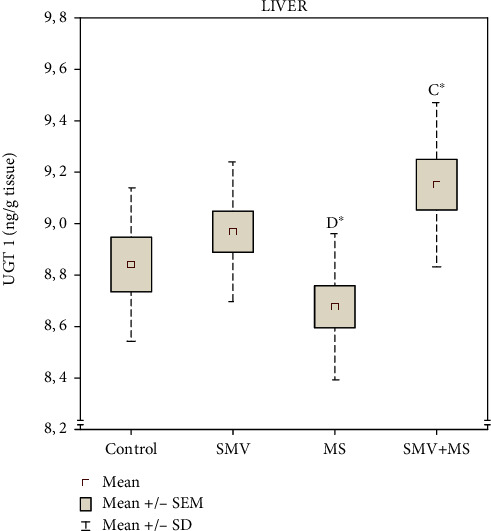
The level of UDP-glucumno-syltransferase 1 (UGT1) in the liver. Differences in the groups are indicated by the following: ^A^versus the control (Group I), ^B^versus the SMV-exposed rats (Group II), ^C^versus the MS-supplemented rats (Group III), and ^D^versus the SMV+MS cosupplied rats (Group IV) (the Tukey HSD test). ^∗^*P* < 0.05.

SMV alone slightly elevated the hepatic UGT1 level, compared to the control (Figure 7). The SMV+MS combination intensified this increase even more, and this level turned out to be significant, in comparison with the MS-supplemented rats, in which the hepatic UGT1 level did not change markedly, compared with the control (Figure 7). As suggested by the two-way ANOVA, the increase in the UGT1 level in the liver of the SMV+MS coadministered animals resulted from the independent action of V and, to a lesser extent, from the trend toward the VxMg interactive action (Table 1). Further, no significant differences between the groups were demonstrated in the hepatic levels of NQO1 and HO-1 (Figures S4 and S5, respectively), and no significant V, Mg, or VxMg effects were indicated by the two-way ANOVA (Table S1).

### 3.7. Correlations between the Measured Variables

Nrf2 positively correlated with GCLC and GSS and with GR, GPx, and GST. Keap1 positively correlated with GCLC, UGT1, and HO-1. Positive correlations were also found between GCLC and GR, GPx, GST, and UGT1 and between GR and GPx, GST, and UGT1. Additionally, GPx positively correlated with GST and UGT1, and GST was positively correlated with MT1 (Table 2). Moreover, MDA displayed a positive correlation with Nrf2, GCLC, and GSS, whereas GCL mRNA positively correlated with GCLC (Table 2). The hepatic V concentration and content (V_Con_ and V_TC_, respectively) were positively correlated to GCLC, GCL mRNA, and UGT1 (Table 2).

Furthermore, there were trends toward positive correlations of Nrf2 with GSH and MT1, Keap1 with GR and GCL mRNA, GCLC with HO-1 and MT1, GCL mRNA with GSS, GR with HO-1 and MDA, GPx with MT1_,_ and UGT1 with HO-1 and MDA (Table 2). There were also tendencies toward positive correlations of V_Con_ with Nrf2 and GSS as well as V_TC_ with Nrf2 and GSS (Table 2). In turn, a significant negative correlation was found between GPx and the hepatic total Mg content (Mg_TC_). Additionally, trends toward negative correlations were observed between GSH and GR as well as between Mg_TC_ and GCLC and GSS (Table 2).

### 3.8. Regression Analysis for Selected Dependent Variables

Based on the regression analysis, the hepatic GCLC, UGT1, GCL mRNA, and MDA levels were significantly correlated with the V dose ingested by the rats throughout the experimental period, whereas the hepatic GR and MT1 levels significantly correlated with the Mg dose consumed by the rats during the same experimental time. Additionally, GSS in the liver tended to correlate with both the V and Mg doses (Table 3).

## 4. Discussion

The present report refers to our previous work, in which the prooxidant potential of V was investigated in the liver of rats receiving SMV alone and in combination with MS [10]. As the interactive effects between V and Mg (supplied simultaneously) were studied with respect to all the parameters measured, it completes our knowledge of the consequences of the VxMg interactive action. The research on VxMg interactions, which have been extensively studied by us at the *in vivo* level [8, 11–13, 32, 47, 48], shows the role played by Mg in V exposure. This issue has not been fully elucidated yet. Certain data on this subject were summarized in a review article by Ścibior [44].

As there are no data on the response of Keap1 and Nrf2 as well as some Nrf2-dependent cytoprotective/detoxifying proteins and MT1 to 12-week SMV and/or MS administration, the discussion is based on our results. The findings reported by other authors, which however fitted the topic of the present report to some degree, have been discussed in this section.

It is well established that OS inactivates Keap1, which leads to release, stabilization, and translocation of Nrf2 to the nucleus, where it regulates the transcription of a variety of cytoprotective genes encoding antioxidant and detoxifying enzymes [49]. In our experimental conditions, the level of the Nrf2 protein significantly increased in the liver of the SMV-exposed rats, compared to the control, which may point to activation of a protective mechanism against V- (SMV-) induced OS reflected by significantly elevated hepatic LPO [10]. In turn, the concentration of Keap1 in the liver of the same group of rats showed an upward trend, compared to the control. In addition, Keap1 positively correlated with Nrf2. In the light of the above, it may be suggested that the increasing trend in the hepatic Keap1 concentration in the SMV-exposed rats may at least in part result from the elevated induction of Keap1 gene expression. Unfortunately, the Keap1 mRNA level has not been investigated in the current study, which makes this statement impossible to confirm at present. To gain better understanding of the Keap1 response to SMV, besides the Keap1 mRNA expression, the level of the p62 protein in the liver of the SMV administered rats will be studied. This will show whether the Keap1 upward trend observed in the liver of the SMV-exposed rats is related to inhibition of Keap1 degradation by p62 accumulation. As known, p62 is able to promote Keap1 degradation in an autophagy-dependent manner. However, when this process is deregulated, both Keap1 and p62 proteins accumulate in the cell [50, 51].

The present study also showed that the expression of GCL in the liver of the SMV-administered rats significantly increased, compared to the control. In addition, the level of the catalytic subunit of GCL (a known rate-limiting enzyme in the de novo GSH biosynthesis pathway) [15], i.e., GCLC (responsible for all the GCL enzymatic activities) [52] also increased, whereas GSS and GSH did not change markedly. The elevated level of the GCL-catalytic (GCLC) subunit, which positively correlated with Nrf2 and GCL mRNA, and the 43% rise in the expression of GCL mRNA clearly indicate increased capacity to synthesize GSH. In turn, the unchanged level of GSS, which positively correlated with Nrf2 and MDA but not with GCLC, may point to a limitation of the second step of GSS-catalyzed GSH biosynthesis during enhanced GCL induction in response to SMV exposure. These findings allow a suggestion that, under 12-week exposure to SMV (0.125 mg V/mL), GCL is intensively induced, but the step in which GSS takes part becomes limited. GSS, which is considered to be of little importance in the regulation of GSH synthesis (according to [52]), has been included in our studies, as elevated GCLC and GSS expression has been reported to enhance GSH synthesis above that found for GCLC alone (according to [52]).

Since OS is associated with elevated GCL activity and GCLC mRNA [15], the increase in the hepatic *GCL* gene expression and GCLC level found in our experimental conditions in response to SMV gives additional evidence for OS development in the liver of rats exposed to SMV. This finding also completes our previous results in this research field [10]. In addition, the elevated concentration and total content of V, demonstrated by us earlier in the liver of rats at SMV administration [32, 47], positively correlated with both the GCLC protein and GCL mRNA. Moreover, the dose of V consumed by the rats throughout the experimental period [32] was positively correlated with GCLC, GCL mRNA, and one of the secondary LPO products formed during OS, i.e., MDA. Furthermore, MDA, whose level increased significantly in the liver of rats exposed to SMV [10], positively correlated with both Nrf2 and GCLC. Some literature data have demonstrated that other cytotoxic aldehydes formed during LPO, i.e., 4-hydroxynonenal (4-HNE), are able to regulate GCLC expression [53, 54]. In addition, Backos et al. [55] have found that 4-HNE can directly modify GCL subunits *in vitro*. Based on these findings and our results, it may be concluded that induction of GCLC is intensified in conditions where increased cellular defense is needed and that SMV activates defense mechanisms in the liver, which are associated with stimulation of GSH synthesis through upregulation of GCL expression.

As far as literature data on the effect of V on the redox-sensitive transcription factor Nrf2 and GCLC are concerned, there is only one paper showing upregulation of mRNA and protein expression of GCLC in response to vanadyl sulfate (VS) in an *in vitro* system where human Chang liver cells were used [56]. The authors of this work found that the treatment of the cells with VS leads to induction of the nuclear translocation of Nrf2 and accumulation of the active form of this protein (phospho-Nrf2). They reported that the induction of GCLC expression by VS is mediated by Nrf2. The authors also explained the signaling pathway involved in VS-mediated Nrf2 activation, suggesting that the higher level of phosphorylated nuclear Nrf2 in the VS treatment is presumably mediated by the phosphorylation activity of activated extracellular regulated kinase (ERK) involved in the nuclear translocation of activated Nrf2 [57]. An increase in ERK phosphorylation after VS treatment noted by the researchers allowed a conclusion that the active phospho form of ERK contributed to the GCLC expression via Nrf2 activation in response to VS. Although these results cannot be directly compared to ours, *in vitro* and *in vivo* studies revealed that the first step in the GSH synthesis is stimulated in response to V. However, in the *in vivo* model, the level of GSH at the SMV administration did not change markedly but increased in an *in vitro* system (where cells were incubated with VS) [56]. These contradictory results obtained from both models are not surprising. It is well-known that such factors as the type of the V compound, valence, dose, duration of treatment/exposure, and sensitivity of cells/organs and organisms determine V response [4]. Since SMV did not significantly change GSS, it would be interesting to know the response of this enzyme to VS. Unfortunately, GSS was not investigated by Kim et al. [56]. In contrast to the present study, a decrease in the GSH concentration previously found by us in the liver of rats at SMV administration [58] may probably be related to methodological differences. Although not determined in our model, an *in vivo* study conducted by Wang et al. [59] showed that the hepatic Nrf2 mRNA expression was downregulated by V in the form of AMV in a high-fat diet.

We also found that the hepatic concentration of the metal binding protein MT1, which is known to be overexpressed in the hepatic tissue in response to heavy metal exposure [29, 60–62], did not markedly change in the liver of the SMV- (0.125 mg V/mL) exposed rats, compared to the control. However, in the liver of the MS- (0.06 mg Mg/mL) supplemented animals, the level of this protein was distinctly (but insignificantly) reduced and further decreased in the liver of the SMV+MS cosupplied rats. As revealed by the two-way ANOVA, this distinct downward trend observed in the SMV+MS cotreated animals only resulted from the independent action of Mg. Since Cu plays a role in the MT synthesis [29], it may be assumed that the lowered concentration and total content of Cu in the liver of the SMV+MS coadministered rats found by us earlier [32, 47] are responsible for the reduced hepatic MT1 level. However, the unchanged hepatic MT1 concentration found in the SMV-exposed rats, in which the lowered hepatic Cu concentration and total Cu content were demonstrated [32, 47], and the unchanged hepatic Cu concentration and total Cu content found in the MS supplemented rats [32, 47], in which the concentration of MT1 showed a visible trend toward a decrease, do not allow us to support this statement. Thus, the mechanism of the Mg-mediated downward trend observed in the hepatic MT1 level in the MS-supplemented and SMV+MS coadministered rats is not clear at present. In the literature, there are single reports showing the influence of Mg or its deficiency on the concentration of MT. For example, a study conducted by Sato et al. [63] provided evidence that Mg injected to mice subcutaneously as magnesium chloride did not affect the hepatic MT level. In turn, studies conducted by Floriańczyk et al. [64] and Kotani et al. [65] revealed a weak positive correlation between Mg and MT as well as increased levels in MT and *MT1* mRNA in the rat liver during Mg deficiency, respectively. Therefore, it would be advisable to examine the MT1 mRNA expression in the rat liver in order to expand our knowledge of the changes in the hepatic MT1 level in the MS-supplemented and SMV+MS cosupplied rats. Furthermore, we showed that the level of MT1 in the liver of rats exposed to SMV (0.125 mg V/mL) remained unchanged, compared to the control. These results may suggest that the hepatic accumulation of V, demonstrated by us previously [32, 47], does not affect the hepatic MT induction. No significant changes in the level of MT in the liver of rats receiving SMV (1.2 mM NaVO_3_/80 mM NaCl) in drinking water were demonstrated by Oster et al. [66]. No marked alterations, compared to the control, in the MT expression in the liver of rats during treatment with V (as ammonium metavanadate, AMV) at a dose of 0.5 ppm in drinking water were observed by Chakraborty et al. [67, 68]. Additionally, Kobayashi et al. [69], who examined the induction of MT in the liver of mice 24 h after subcutaneous administration of AMV at doses of 50, 100, 200, or 300 *μ*mol/kg, found that the level of MT in the liver, in which the accumulation of V was very low, increased by AMV injection in a dose-dependent manner [69].

Further, we also revealed that the elevated hepatic MDA level in the SMV and SMV+MS coadministered rats [10] was accompanied by the visible upward trends in the hepatic levels of some of cytoprotective/detoxifying enzymes. For example, GST tended to be elevated in the liver of the SMV-exposed rats, in which the hepatic LPO was intensified [10]. These changes may point to activation of mechanisms of detoxification of electrophilic aldehydic LPO products, as GSTs play an important role in the regulation of intracellular concentrations of compounds generated during the free radical process [70]. It is well-known that the OS-induced LPO products such as 4-HNE and MDA are metabolized via GST-mediated conjugation and exported from the cell [71]. Tjalkens et al. [72], who demonstrated induction of certain GST isoenzymes in response to aldehydic products generated by hepatic LPO in a murine model, confirmed the role of GST in the protective mechanism against LPO. Enhanced LPO was suggested as a factor underlying vanadate toxicity by Younes and Strubelt [73], who found a strong correlation between induction of LPO and hepatotoxicity. The authors investigated the toxic potential of vanadate towards isolated perfused rat livers. Elevated LPO was also highlighted as one of the determinants of vanadate toxicity by Elfant and Keen [74] based on the results from *in vivo* studies. Interestingly, in our rodent model, GST, GCLC, GR, and GPx positively correlated with each other and with Nrf2; in turn, GSH and Nrf2 correlated more weakly. Furthermore, UGT, which is highly expressed in the liver [75], showed a distinct upward trend in the liver of rats receiving SMV and MS in combination, and this effect was not only related to the independent action of V but also resulted from the trend toward the VxMg interactive action. In addition, these changes were positively correlated with the hepatic V concentration and content and with the hepatic GR and GPx levels. Based on these findings, we may conclude that certain components of antioxidant defense and some phase II metabolizing proteins that detoxify both exogenous and endogenous compounds cooperate in the conditions of the SMV and MS administration. To gain better insight into the observed changes, the *Nrf2 mRNA* expression and the expression of genes for GST, GPx, GR, and UGT1 will be analyzed in future studies. This type of research may provide a new outlook for analysis of the genetic enhancement of protection of the liver against potential oxidative damage in response to SMV. It would be interesting to know possible differences in the levels of expression of mRNA for GST, GPx, GR, and UGT1 as well as the ratios between them. As shown by the literature data, there are no reports on this issue, although cellular defense mechanisms have been investigated in the rodent liver using different V compounds, V concentrations/doses, and route and/or time of V administration [36, 66, 74, 76–78].

**Figure 8:**
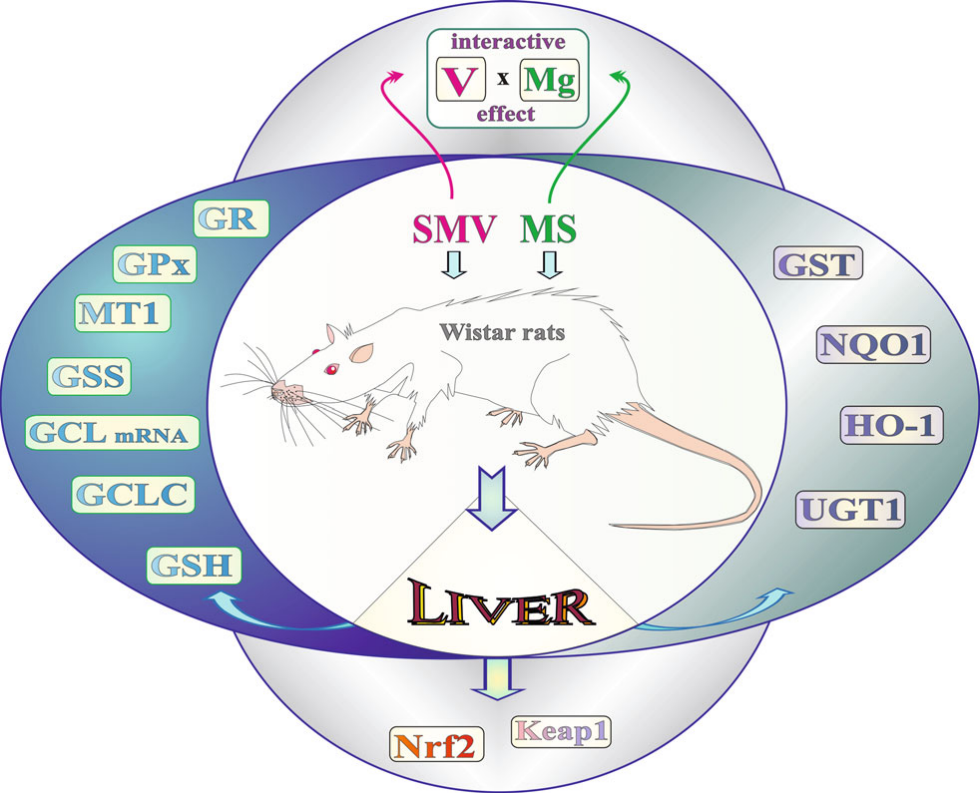
A graphical summary of indices included in the study.

As for NQO1 and HO-1, neither SMV (0.125 mg V/mL) nor MS (0.06 mg Mg/mL) and their combination (SMV+MS) caused significant changes in the levels of these proteins in the liver, compared to the control. Studies conducted by other authors on the effect of V on NQO1 and HO-1 are provided below, although they are not directly comparable to our results. For example, Sánchez-González et al. [79] demonstrated a decrease in the NQO1 activity in the liver of diabetic rats following treatment with 3 mg V/day of bis(maltolato)oxovanadium IV (BMOV), compared to untreated diabetic animals, in which the hepatic NQO1 activity significantly increased, in comparison with the control. As highlighted, a rise in the NQO1 activity in the liver of diabetic rats may favor a decrease in the formation of ROS, as NQO1 offers protection against the toxic effects of oxidants, heavy metals, and carcinogens [21, 80, 81]. Other authors found that high-fat diet enhanced a V-induced decrease in the hepatic NQO1 activity and NQO1 mRNA expression [59]; in this case, V was administered as AMV (15 and 30 mg V/kg). In turn, *in vitro* studies with HepG2 cells demonstrated that V (as AMV) did not markedly change the NQO1 activity at the concentration of 25 *μ*M, but led to a significant decrease in the activity of NQO1 and NQO1 mRNA expression at higher concentrations (i.e., 50, 100, and 250 *μ*M) [82]. Similarly, studies with murine hepatoma cells (Hepa 1c1c7) also showed that V (as AMV) supplied at the above-mentioned concentrations reduced the mRNA expression of NQO1 and the NQO1 activity [83]. As far as HO-1 is concerned, studies showed increased HO-1 activity in the liver of C57BL/6 mice receiving V- (26 *μ*g/L) containing Jeju water for 90 days [84] and in human Chang liver cells following incubation with the same Jeju water [85]. In turn, a study conducted by Abdelhamid et al. [86], who investigated the effect of AMV on the level of HO-1 mRNA in the human hepatoma HepG2 cells treated with increasing concentrations of V (25-250 *μ*M), showed that V did not significantly alter HO-1 mRNA. The parameters included in the study are graphically summarized in Figure 8.

The limitation of this study is that we have not yet examined the exact underlying mechanism of the Mg-independent action and the VxMg interactive effect. This aspect will be the subject of a new study, which will constitute a separate article.

## 5. Conclusions

For the first time, our findings provided insight into the response of the Nrf2/Keap1 system, GCL mRNA expression, some cytoprotective/detoxifying proteins, and MT1 to the 12-week V and/or Mg administration in the form of SMV and MS, respectively, in the rat liver. The following conclusions can be formulated: (a) the increased hepatic Nrf2 level in the SMV-receiving rats confirms the presence of OS in the liver for which enhanced LPO and V accumulation were previously demonstrated [10, 32, 47]; (b) the elevated hepatic GCL mRNA expression and the GCLC level as well as the unchanged hepatic GSS and GSH levels indicate the capacity to synthesize GSH and simultaneously suggest a limitation of the second step in GSH synthesis at the SMV exposure; (c) the upward trend in the level of the hepatic Keap1 protein points to a need to determine the p62 protein, which might be responsible for the inhibition of Keap1 degradation at SMV administration; (d) the positive correlations revealed between certain cytoprotective/detoxifying proteins and between them and indices (examined by us previously) [10, 32, 47] such as MDA, hepatic V concentration/total content, and/or V dose, point to cooperation between the components of antioxidant defense in the conditions of hepatic V accumulation and LPO intensification; (e) the mechanism of the Mg-independent action involved in changes in the hepatic MT1 concentration is not clear at present and deserves further exploration; (f) further studies are also needed to elucidate the mechanisms of the VxMg interactive effect and trend in changes in the levels of the Nrf2 and UGT1 proteins, respectively, in the liver of the SMV+MS cosupplied rats; (g) investigations of the Nrf2, Keap1, GST, GPx, GR, GSS, and UGT1 mRNA expression in the liver of rats receiving SMV separately and in combination with MS will not only ensure better understanding of the observed changes at SMV and MS administration but also provide a new outlook for analysis of the genetic protection of liver against the adverse SMV action.

## Figures and Tables

**Table 1:** V and Mg main and interactive effects on selected parameters in male Wistar rats after combined administration of SMV and MS.

Variables	Two-way ANOVA analysis		
	Main effect of V	Main effect of Mg	Interactive effect (VxMg)
Nrf2	NS	NS	*F* = 7.66, *P* < 0.01
Keap1	NS	NS	NS
GSH	NS	NS	NS
GCLC	*F* = 16.97, *P* < 0.001	NS	NS
GCL mRNA	*F* = 28.531, *P* < 0.0001	NS	NS
GSS	*F* = 2.89, *P* = 0.096	*F* = 3.93, *P* = 0.053	NS
MT1	NS	*F* = 4.129, *P* < 0.01	NS
UGT1	*F* = 8.14, *P* < 0.01	NS	*F* = 2.96, *P* = 0.091

Nrf2: nuclear factor erythroid-derived 2-related factor 2 (*μ*g/g); Keap1: Kelch-like ECH-associated protein 1 (*μ*g/g); GSH: reduced glutathione (*μ*g/g); GCLC: glutamate cysteine ligase catalytic subunit (ng/g); GCL mRNA: glutamate cysteine ligase gene expression; GSS: glutathione synthetase (U/g); MT1: metallothionein-1 (ng/g); UGT1: UDP-glucumno-syltransferase 1 (ng/g). Data are presented as *F* values and the levels of significance (*P*). NS: no statistically significant effect.

**Table 2:** Correlation coefficients for compared variables.

Variables^a^	Nrf2	Keap1	GCLC	GSS	GSH	GR	GPx	GST	UGT1	NQO1	HO-1	MT1	GCL mRNA	MDA^b^
Nrf2	1													
Keap1	§	1												
GCLC	0.337^∗^	0.460^†^	1											
GSS	0.467^†^	§	§	1										
GSH	*0.248* ^#k^	§	§	§	1									
GR	0.295^∗^	*0.265* ^#f^	0.642^‡^	§	-0.270^#e^	1								
GPx	0.412^†^	§	0.394^†^	§	§	0.509^‡^	1							
GST	0.405^†^	§	0.445^†^	§	§	0.525^‡^	0.482^‡^	1						
UGT1	§	0.308^∗^	0.561^‡^	§	§	0.286^∗^	0.309^∗^	§	1					
NQO1	§	§	§	§	§	§	§	§	§	1				
HO-1	§	0.330^∗^	*0.257* ^#i^	§	§	*0.249* ^#l^	§	§	*0.257* ^#g^	§	1			
MT1	*0.255* ^#j^	§	*0.260* ^#k^	§	§	§	*0.270* ^#d^	0.436^†^	§	§	§	1		
GCL mRNA	§	*0.242* ^#p^	0.323^∗^	*0.260* ^#n^	§	§	§	§	§	§	§	§	1	
MDA^b^	0.291^∗^	§	0.484^‡^	0.307^∗^	§	*0.256* ^#h^	§	§	*0.270* ^#b^	§	§	§	§	1
Variables^a^	V_Con_^c^					V_TC_^d^					Mg_TC_^d^			
Nrf2	*0.243* ^#k^					*0.231* ^#o^					§			
GCLC	0.463^†^					0.420^†^					*-0.254* ^#i^			
GCL mRNA	0.544^‡^					0.540^‡^					§			
GSS	*0.274* ^#c^					*0.277* ^#a^					*-0.244* ^#m^			
GPx ^ƒ^	§					§					-0.285^∗^			
UGT1	0.331^∗^					0.305^∗^					§			

Data are presented as correlation coefficients (*r*) and levels of statistical significance (*P*). Significant correlations and tendencies toward them are highlighted as normal text and italics, respectively. ^a^Nrf2: nuclear factor erythroid-derived 2-related factor 2 (*μ*g/g); Keap1: Kelch-like ECH-associated protein 1 (*μ*g/g); GCLC: glutamate cysteine ligase catalytic subunit (ng/g); GSS: glutathione synthetase (U/g); GSH: reduced glutathione (*μ*g/g); GR: glutathione reductase (pg/g); GPx: glutathione peroxidase (ng/g); GST: glutathione S-transferase (ng/g); UGT1: UDP-glucumno-syltransferase 1 (ng/g); NQO1: NAD(P)H dehydrogenase (quinone 1) (ng/g); HO-1: heme oxygenase 1 (*μ*g/g); MT1: metallothionein-1 (ng/g); GCL mRNA: glutamate cysteine ligase gene expression; MDA: malondialdehyde (nM/g tissue); V_Con_: concentration of vanadium in the liver (*μ*g/g); V_TC_: total content of vanadium in the liver; Mg_TC_: total content of magnesium in the liver. ^§^Lack of a linear relationship. ^ƒ^Logarithmically transformed data. ^‡^*P* < 0.001; ^†^*P* < 0.01; ^∗^*P* < 0.05. ^#a^*P* = 0.052, ^#b^*P* = 0.053, ^#c^*P* = 0.054, ^#d^*P* = 0.055, ^#e^*P* = 0.056, ^#f^*P* = 0.058, ^#g^*P* = 0.066, ^#h^*P* = 0.067, ^#i^*P* = 0.072, ^#j^*P* = 0.073, ^#k^*P* = 0.074, ^#l^*P* = 0.075, ^#m^*P* = 0.081, ^#n^*P* = 0.085, ^#o^*P* = 0.090, ^#p^*P* = 0.091. Published previously: ^b^[10, 11], ^c^[32], and ^d^[47].

**Table 3:** Summary of the linear regression analysis for selected dependent variables.

Variables^a^	Statistics (ANOVA) *F*(regression df, residual df) = mean square; significance	Predictors	
		V_D_^b^ (consumed with d.w.)	Mg_D_^b^ (consumed with d.w.)
GCLC	*F*(2, 48) = 106.355, *P* < 0.001	*β* = 0.531, *P* < 0.001	*β* = 0.076, NS
GCL mRNA	*F*(2, 49) = 0.414, *P* < 0.001	*β* = 0.581, *P* < 0.001	*β* = 0.065, NS
GSS	*F*(2, 47) = 1548.187, *P* < 0.05	*β* = 0.237, *P* = 0.089	*β* = −0.258, *P* = 0.065
GR^ƒ^	*F*(2, 50) = 0.024, *P* < 0.05	*β* = 0.199, NS	*β* = 0.302, *P* < 0.05
UGT1	*F*(2, 50) = 0.610, *P* < 0.05	*β* = 0.391, *P* < 0.01	*β* = 0.030, NS
MT1	*F*(2, 49) = 296.708, *P* < 0.05	*β* = −0.080, NS	*β* = −0.386, *P* < 0.01
MDA^c^	*F*(2, 53) = 33806.493, *P* < 0.01	*β* = 0.471, *P* < 0.001	*β* = 0.053, NS

^a^GCLC: glutamate cysteine ligase catalytic subunit (ng/g); GSS: glutathione synthetase (U/g); GR: glutathione reductase (pg/g); UGT1: UDP-glucumno-syltransferase 1 (ng/g); MT1: metallothionein-1 (ng/g); GCL mRNA: glutamate cysteine ligase gene expression; MDA: malondialdehyde (nM/g tissue); V_D_: vanadium dose (mg V/kg b.wt./24 h); Mg_D_: magnesium dose (mg Mg/kg b.wt./24 h). NS: not statistically significant; d.w.: drinking water; df: degrees of freedom; *β*: standardized coefficient (beta); *P*: level of significance. ^ƒ^Logarithmically transformed data. Published previously: ^b^[32] and ^c^[10, 11].

## Data Availability

Data are available on request.

## Abbreviations

AMV: Ammonium metavanadate
b.wt.: Body weight
DEPC: Diethylpyrocarbonate
DPBS: Dulbecco's phosphate-buffered saline
d.w.: Drinking water
ELISA: Enzyme-linked immunosorbent assay
ERK: Extracellular regulated kinase
GCLC: Glutamate cysteine ligase catalytic subunit
GCL: Glutamate-cysteine ligase
GPx: Glutathione peroxidase
GR: Glutathione reductase
GSH: Reduced glutathione
GSS: Glutathione synthetase
GST: Glutathione S-transferase
Hepa 1c1c7: Murine hepatoma cells
HepG2: Human liver cancer cell line
4-HNE: 4-Hydroxynonenal
HO-1: Heme oxygenase
Keap1: Kelch-like ECH-associated protein 1
LPO: Lipid peroxidation
MDA: Malondialdehyde
Mg: Magnesium
Mg_D_: Magnesium dose
Mg_TC_: Total content of magnesium
MS: Magnesium sulfate
MT1: Metallothionein-1
MTs: Metallothioneins
Nrf2: Nuclear factor erythroid-derived 2-related factor 2
NQO1: NAD(P)H dehydrogenase quinone 1
OS: Oxidative stress
PCR: Polymerase chain reaction
RBC: Erythrocytes
ROS: Reactive oxygen species
RT: Reverse transcription
SD: Standard deviation
Sdh: Succinate dehydrogenase complex
SEM: Standard error of the mean
SMV: Sodium metavanadate
UGT1: UDP-glucumno-syltransferase 1
V: Vanadium
V_Con_: Concentration of vanadium
V_D_: Vanadium dose
V_TC_: Total content of vanadium
VS: Vanadyl sulfate
VxMg: Vanadium-magnesium interaction.

## Supplementary Materials

Supplementary 1 Table S1: V and Mg main and interactive effects on glutathione reductase (GR), glutathione peroxidase (GPx), glutathione S-transferase (GST), NAD(P)H dehydrogenase quinone 1 (NQO1), and heme oxygenase 1 (HO-1) in male Wistar rats after combined administration of SMV and MS.

Supplementary 2 Figure S1: The level of glutathione reductase (GR) in the liver.

Supplementary 3 Figure S2: The level of glutathione peroxidase (GPx) in the liver.

Supplementary 4 Figure S3: The level of glutathione S-transferase (GST) in the liver.

Supplementary 5 Figure S4: The level of NAD(P)H dehydrogenase quinone 1 (NQO1) in the liver.

Supplementary 6 Figure S5: The level of heme oxygenase 1 (HO-1) in the liver.
