# Supplementary material for: Response of Cytoprotective and Detoxifying Proteins to Vanadate and/or Magnesium in the Rat Liver: The Nrf2-Keap1 System
Source: Oxid Med Cell Longev. 2021 Dec 13;2021:8447456. doi: 10.1155/2021/8447456 (PMC8689234; doi:10.1155/2021/8447456)
Supplement: Supplementary 1 — Table S1: V and Mg main and interactive effects on glutathione reductase (GR), glutathione peroxidase (GPx), glutathione S-transferase (GST), NAD(P)H dehydrogenase quinone 1 (NQO1), and heme oxygenase 1 (HO-1) in male Wistar rats after combined administration of SMV and MS. [file 8447456.f1.doc]

**Supplementary Materials: Table S1**

**Table S1.** V and Mg main and interactive effects on glutathione reductase (GR), glutathione peroxidase (GPx), glutathione S-transferase (GST), NAD(P)H dehydrogenase quinone 1 (NQO1), and heme oxygenase 1 (HO-1) in male Wistar rats after combined administration of SMV and MS.

| Two-way ANOVA analysis | |
| --- | --- |
| GR^‡^ | |
| Main effect of V | NS |
| Main effect of Mg | F = 4.9, *P <* 0.05 |
| Interactive effect (VxMg) | NS |
| GPx^‡^ | |
| Main effect of V | NS |
| Main effect of Mg | NS |
| Interactive effect (VxMg) | NS |
| GST^‡^ | |
| Main effect of V | NS |
| Main effect of Mg | NS |
| Interactive effect (VxMg) | NS |
| NQO1 | |
| Main effect of V | NS |
| Main effect of Mg | NS |
| Interactive effect (VxMg) | NS |
| HO-1 | |
| Main effect of V | NS |
| Main effect of Mg | NS |
| Interactive effect (VxMg) | NS |

Data are presented as F values and the levels of significance (*P*). NS: no statistically significant effect.

^‡^ Logarithmically transformed data.
