# Supplementary figures and images for: Response of Cytoprotective and Detoxifying Proteins to Vanadate and/or Magnesium in the Rat Liver: The Nrf2-Keap1 System
Source: Oxid Med Cell Longev. 2021 Dec 13;2021:8447456. doi: 10.1155/2021/8447456 (PMC8689234; doi:10.1155/2021/8447456)

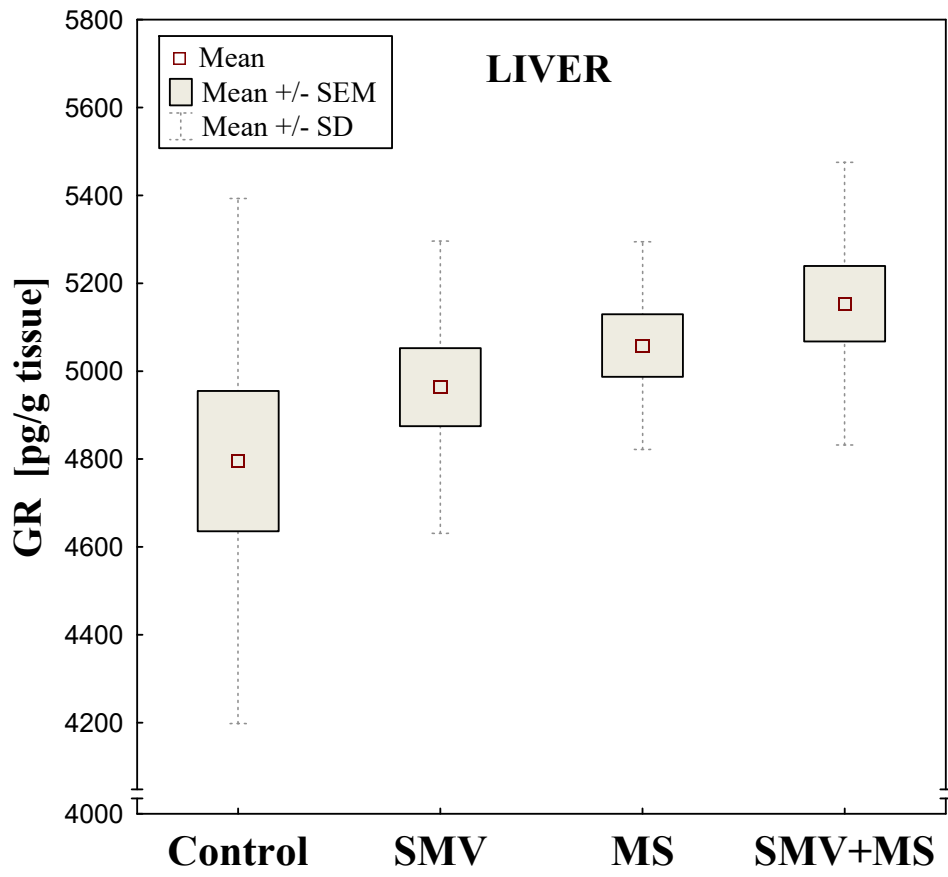

Supplement: Supplementary 2 — Figure S1: The level of glutathione reductase (GR) in the liver. [file 8447456.f2.pdf]

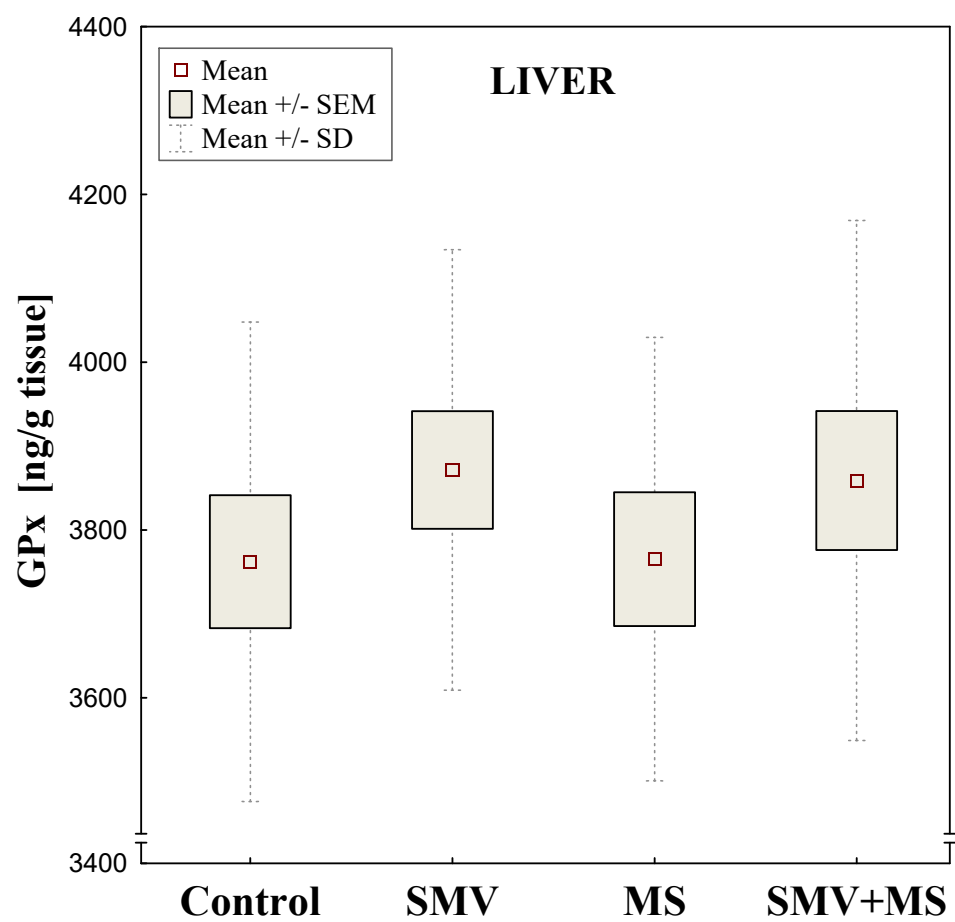

Supplement: Supplementary 3 — Figure S2: The level of glutathione peroxidase (GPx) in the liver. [file 8447456.f3.pdf]

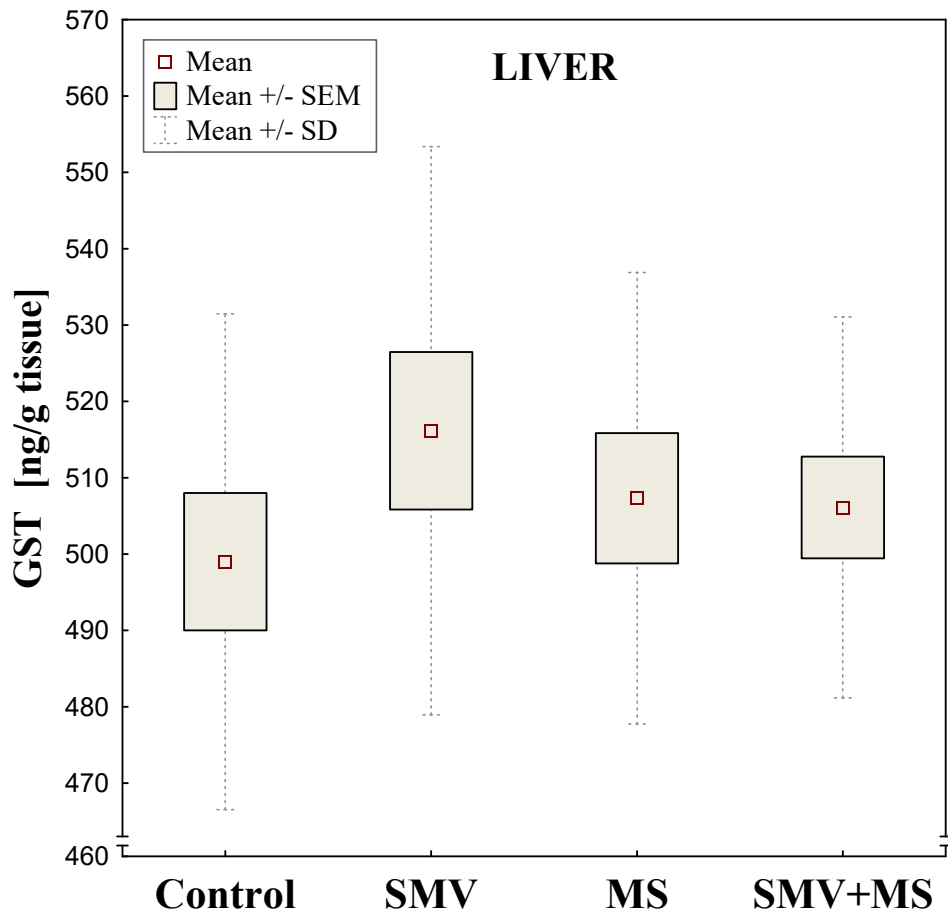

Supplement: Supplementary 4 — Figure S3: The level of glutathione S-transferase (GST) in the liver. [file 8447456.f4.pdf]

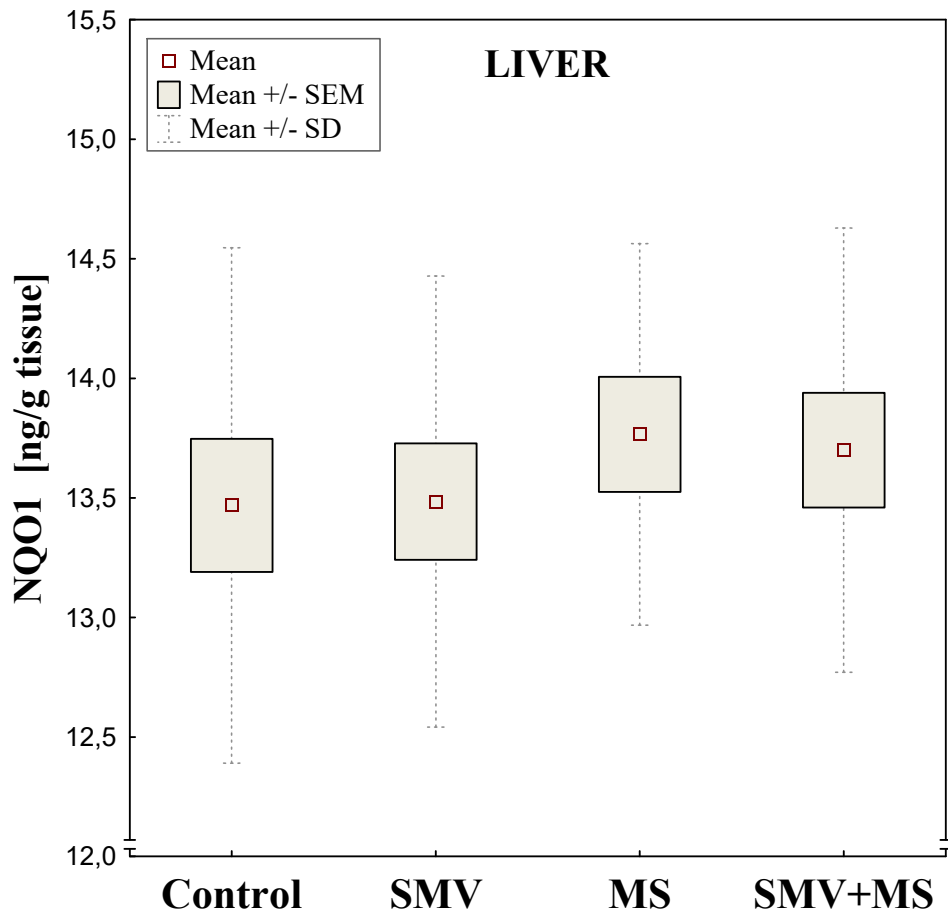

Supplement: Supplementary 5 — Figure S4: The level of NAD(P)H dehydrogenase quinone 1 (NQO1) in the liver. [file 8447456.f5.pdf]

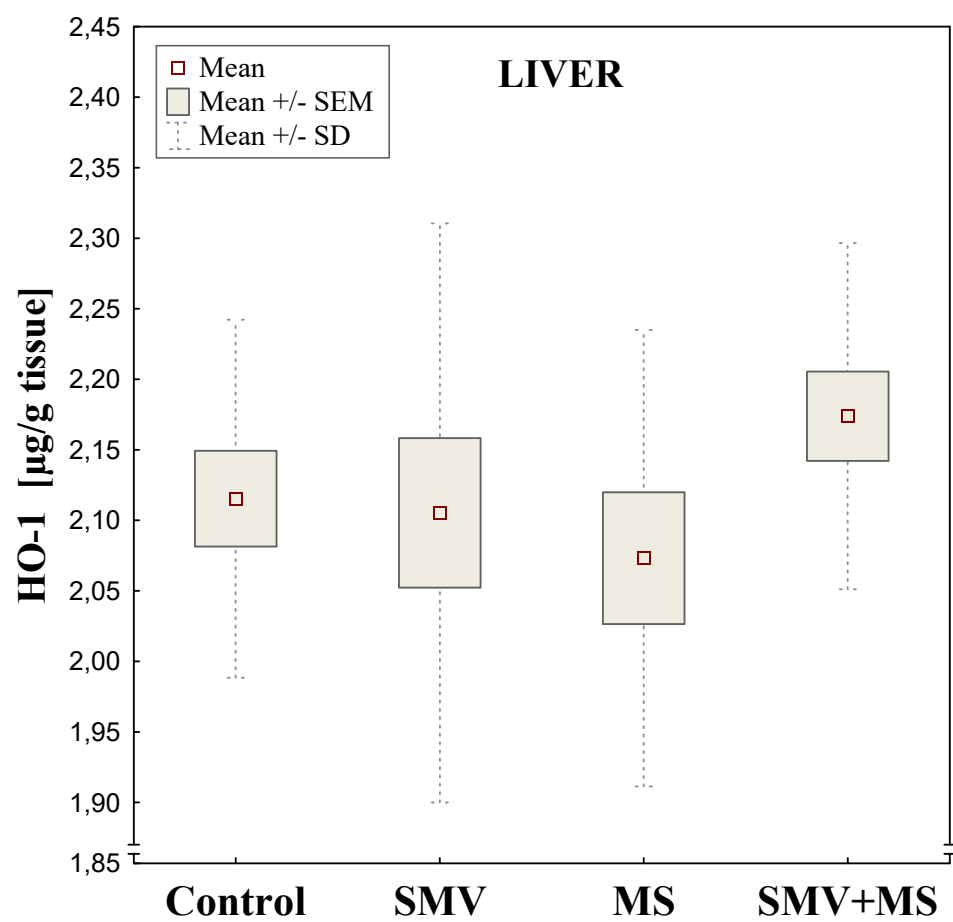

Supplement: Supplementary 6 — Figure S5: The level of heme oxygenase 1 (HO-1) in the liver. [file 8447456.f6.pdf]
